# Prevalence of depression, anxiety and post-traumatic stress in war- and conflict-afflicted areas: A meta-analysis

**DOI:** 10.3389/fpsyt.2022.978703

**Published:** 2022-09-16

**Authors:** Isis Claire Z. Y. Lim, Wilson W. S. Tam, Agata Chudzicka-Czupała, Roger S. McIntyre, Kayla M. Teopiz, Roger C. Ho, Cyrus S. H. Ho

**Affiliations:** ^1^Department of Psychological Medicine, Yong Loo Lin School of Medicine, National University of Singapore, Singapore, Singapore; ^2^Alice Lee School of Nursing, Yong Loo Lin School of Medicine, National University of Singapore, Singapore, Singapore; ^3^Faculty of Psychology, SWPS University of Social Sciences and Humanities, Katowice, Poland; ^4^Mood Disorders Psychopharmacology Unit, University Health Network, Toronto, ON, Canada; ^5^University of Toronto, Toronto, ON, Canada; ^6^Brain and Cognition Discovery Foundation, Toronto, ON, Canada; ^7^Braxia Scientific Corp., Toronto, ON, Canada; ^8^Institute for Health Innovation and Technology (iHealthtech), National University of Singapore, Singapore, Singapore; ^9^Department of Psychological Medicine, National University Health System, Singapore, Singapore

**Keywords:** depression, anxiety, post-traumatic stress, mental illness, mental health, conflict, war, post-war

## Abstract

**Background:**

With the rise of fragility, conflict and violence (FCV), understanding the prevalence and risk factors associated with mental disorders is beneficial to direct aid to vulnerable groups. To better understand mental disorders depending on the population and the timeframe, we performed a systematic review to investigate the aggregate prevalence of depression, anxiety and post-traumatic stress symptoms among both civilian and military population exposed to war.

**Methods:**

We used MEDLINE (PubMed), Web of Science, PsycINFO, and Embase to identify studies published from inception or 1–Jan, 1945 (whichever earlier), to 31–May, 2022, to reporting on the prevalence of depression, anxiety and post-traumatic stress symptoms using structured clinical interviews and validated questionnaires as well as variables known to be associated with prevalence to perform meta-regression. We then used random-effects bivariate meta-analysis models to estimate the aggregate prevalence rate.

**Results:**

The aggregate prevalence of depression, anxiety and post-traumatic stress during times of conflict or war were 28.9, 30.7, and 23.5%, respectively. Our results indicate a significant difference in the levels of depression and anxiety, but not post-traumatic stress, between the civilian group and the military group respectively (depression 34.7 vs 21.1%, *p* < 0.001; anxiety 38.6 vs 16.2%, *p* < 0.001; post-traumatic stress: 25.7 vs 21.3%, *p* = 0.256). The aggregate prevalence of depression during the wars was 38.7% (95% CI: 30.0–48.3, *I*^2^ = 98.1%), while the aggregate prevalence of depression post-wars was 29.1% (95% CI: 24.7–33.9, *I*^2^ = 99.2%). The aggregate prevalence of anxiety during the wars was 43.4% (95% CI: 27.5–60.7, *I*^2^ = 98.6%), while the aggregate prevalence of anxiety post-wars was 30.3% (95% CI: 24.5–36.9, *I*^2^ = 99.2%). The subgroup analysis showed significant difference in prevalence of depression, and anxiety between the civilians and military group (*p* < 0.001).

**Conclusion:**

The aggregate prevalence of depression, anxiety and post-traumatic stress in populations experiencing FCV are 28.9, 30.7, and 23.5%, respectively. There is a significant difference in prevalence of depression and anxiety between civilians and the military personnels. Our results show that there is a significant difference in the prevalence of depression and anxiety among individuals in areas affected by FCV during the wars compared to after the wars. Overall, these results highlight that mental health in times of conflict is a public health issue that cannot be ignored, and that appropriate aid made available to at risk populations can reduce the prevalence of psychiatric symptoms during time of FCV.

**Systematic Review Registration:**

https://www.crd.york.ac.uk/prospero/display_record.php?RecordID=337486, Identifier 337486.

## Introduction

With the rise of conflict and violence (FCV) in places like Sudan, Somalia, and Ukraine, the World Bank estimates that a total of 82.4 million people were forcibly displaced as of end-2020, a sharp increase from the estimated 68.5 million in 2017 (1, 2). War is defined as organized violence where violence is the primary means of coercion to achieve the continuation of a group's policy; the violence may target individuals or resources, but it is always physical and extends beyond the nation-state (3). As war-afflicted areas are often associated with higher levels of psychosocial distress and increases the susceptibility of a population to psychiatric symptoms, there has been growing interest in the psychosocial health of persons in war-afflicted areas (4).

The effects of wars on mental health, physical health, economic security, and political stability are long-lasting. A systematic review on long-settled refugees estimated the prevalence of any psychiatric morbidity to be about 20% in a population that has resettled for at least 5 years, and acknowledges risk factors predicting higher rates of psychiatric symptoms such as post-traumatic stress and the adverse socio-economic situation (5, 6). This is further fueled by the recent highly reported war in Ukraine which saw a rise in displaced individuals and separated families, raising global awareness for mental wellness during times of armed conflict (7). The cumulative effects of the Ukraine war are likely to predispose its civilians and military to adverse mental health outcomes due to rapid transformations of their lives, such as civilians taking up volunteer military roles, or being exposed to trauma (8). Along with the added stressor of the COVID-19 pandemic, the Russian invasion of Ukraine saw a surge of mental health disorders along with a reduction in mental healthcare-seeking behavior, highlighting the need to underscore the importance of ramping up the accessibility of mental health aids especially in times of conflict (9, 10). This is congruent with a recent meta-analysis that reported the prevalence of mental disorders post-conflict, but was limited in its assessment of psychiatric symptoms during the time of conflict itself. Our analysis aims to highlight the well-established link between FCV and mental health disorders, and underscore the importance of providing appropriate aid to populations affected by conflict (11).

The military, being at the forefront of armed conflict, is often believed to be at higher risk of experiencing psychiatric symptoms due to increased combat exposure leading to psychological distress (12, 13). It has been separately reported that civilians are often the overwhelming survivors of war trauma, and are vulnerable subjects to the aftermath effects of war (14, 15). Previous studies have reported on either the civilian or military population, but rarely both (16). By comparing both these groups, this analysis can provide meaningful insights on the types of interventions, exposures, and perpetuating factors of psychiatric symptoms in the context of FCVs. Furthermore, the risk factors and maintenance factors of psychiatric symptoms during wars and post-wars may differ and hence affect their prevalence. Epidemiological studies on this topic are notoriously subjected to large heterogeneity owing to the method of sampling that was implemented, the severity of the conflict and country in which the sampling was done (11, 17). The foregoing limitations in previous research makes interpretation and estimation of global prevalence of psychiatric symptoms related to FCV challenging (18).

Many studies have documented a population's war-related mental suffering and estimated the manifestations of mental health disorders caused by armed conflict, but there is a lack of research that has focused on comparing the severity of mental health disorder symptoms experienced by military and civilians. Our study aims to fill this gap in research. This seems particularly important in the face of data of The United Nations Security Council that stresses that 90% of the victims of war are civilians, innocent people, who should be especially protected during wartime conflicts (19). Making a comparison of the negative mental health effects resulting from participation in armed conflict between civilians and trained soldiers may provide a deeper insight into the types of symptoms and their severity in both groups. We performed a systematic review to investigate the aggregate prevalence of depression, anxiety and post-traumatic stress among both civilian and military populations exposed to war, and better understand the susceptibility to or permeance of psychiatric symptoms depending on the populations and the timeframe with reference to the given war. We aimed to address the heterogeneity by using a random effects model because the weight given to each study would be less influenced by sample size, followed by performing appropriate subgroup analyses and meta-regressions (20, 21).

## Methods

### Search strategy

The meta-analysis was reported according to the Preferred Reporting Items of Systematic Reviews and Meta-Analyses (PRISMA) guidelines (22). The protocol for this study was registered and is under open access by the International Prospective Register of Systematic Reviews (PROSPERO). We used MEDLINE (PubMed) to identify studies published from January 1, 1945, to May 31, 2022 and other electronic databases such as Web of Science, PsycINFO, and Embase from inception to 31 May, 2022, to identify articles study prevalence of depression, anxiety and post-traumatic stress based on structured clinical assessment or questionnaires in people exposed to FCV. We used the search strategy {[“war” (All Fields)] AND [“mental health” (MeSH) OR “mental disorders” (MeSH) OR “depression” (MeSH) OR “depressive disorder” (MeSH) OR “depression” (MeSH) OR “anxiety disorder” (MeSH) OR “anxiety” (MeSH) OR “PTSD” (MeSH) OR “post-traumatic stress disorder” (MeSH) OR “psychological impact” (MeSH) OR” post-traumatic stress disorder” (MeSH)]} to search for articles using PubMed, and identified further sources using the reference lists from studies such as systematic reviews from articles obtained through the initial search. We included all studies (e.g., randomized cohort trials, retrospective/prospective cohort studies, cross-sectional study) according to the PICOS (Table 1). The literature search and data extraction were performed independently by two reviewers. Quality control was performed by two independent reviewers with the modified Newcastle-Ottawa Scale to assess the risk of bias in observational studies, and all emerging conflicts were resolved by consensus (23).

**Table 1:** PICOS, inclusion criteria and exclusion criteria applied to database search.

**PICOS**	**Inclusion criteria**	**Exclusion criteria**
Population	Military (Army, Navy, Air Force, Marines, Coast Guard, medics, and Reservists/National Guard), civilians, refugees, prisoners-of-war from countries directly involved in war Population in countries of war and conflict (direct organized armed violence, terrorism, insurgence, espionage etc), where violence is the one of the means of coercion	2nd or 3rd generation survivors of war, civilians not from countries directly involved in war, non-deployed military, pregnant cohort, cohorts comparing medical conditions (skewed cohorts)
Intervention	–	Population that only underwent psychiatric interventions
Comparison	Military and civilian During, post-war (3-months after conflict official end date)	–
Outcome	Depression, anxiety, post-traumatic stress The outcomes of interest were depression, anxiety and PTSD, but studies containing any one of depression, anxiety, PTSD, or substance or alcohol misuse were included to ensure that no studies reporting the psychiatric symptoms of interest were missed. The study provided enough information to generate an odds ratio (OR) by subgroups.	Combat anxiety, alcohol or substance use disorders The sample was based on clinical or injured or treatment/help-seeking population/s, including studies based on data from Veterans Affairs (VA) treatment facilities.
Study designs	Clinical trials, randomized controlled trials, cross sectional, case control, retrospective outcome study, prospective study Published or translated in English	–

### Inclusion and exclusion criteria

The inclusion criteria for studies eligible for analysis were as follows: (1) Population studied of either military (Army, Navy, Air Force, Marines, Coast Guard, medics, and Reservists/National Guard), civilians, refugees, prisoners-of-war from countries directly involved in war and conflict where violence is one of the means of coercion; (2) The outcomes of interest were depression, anxiety and post-traumatic stress; (3) The aforementioned psychiatric symptoms were assessed by structured clinical interviews or questionnaires. (4) Populations were defined as military if the targeted study population stated that they were from a military background such as but not limited to Army, Navy, Air Force, Marine, Coast Guard, National Guard, Veterans, Prisoner-of-war; whereas a population was defined as civilian if it were studying the general population, civilian or refugees. (5) A study was considered as during war if the data collection occurred during the time of conflict; a study was considered as post-war if the data was collected at least 4 months after the official end date of the conflict, or if the direct stressor (exposure to war) had been removed (e.g., in refugees population).

The exclusion criteria for the studies included: (1) Population studied were the second or third generation survivors of war, civilians not from countries directly involved in war, non-deployed military, pregnant cohort, cohorts comparing medical conditions (skewed cohorts); (2) all participants received mental health interventions (part of a randomized control trial).

### Data analysis

We used the Comprehensive Meta-Analysis (CMA) Version 3.0 (Biostat, Inc., Englewood, NJ, USA) to perform all statistical analyses. A random effects meta-analysis was conducted to investigate the prevalence of depression, anxiety and post-traumatic stress. The random-effects model was utilized to account for between-study variance (24).

Prevalence of the condition were reported as a dichotomous variable (i.e., presence vs absence) according to the assessments established by structured clinical interviews or questionnaires. Forest plots for the prevalence of each psychiatric condition overall and within subgroups were made to represent the overall estimate, as well as individual study estimates. Thus, the aggregate prevalence of each outcome (i.e., depression, anxiety and post-traumatic stress), its corresponding *p*-value and 95% confidence interval (CI) were reported. A *p*-value < 0.05 was considered significant. The *I*^2^ statistic was used to assess heterogeneity among studies. As a reference, *I*^2^ values of 25% are considered low, those of 50% are moderate, and those of 75% are high (25). Meta-regression was performed to examine the impact of moderator variables of mean age and the female sex on the study effect size when significant heterogeneity was detected, using a random-effects model. Other moderator variables were not possible to analyze due to limited data availability. The potential for publication bias was inspected visually using the funnel plot and Egger's regression method. In the event that publication bias was detected, the fill and trim test would be performed to establish the potential number of missing studies.

## Results

### Selection and inclusion of studies

**Figure 1:**
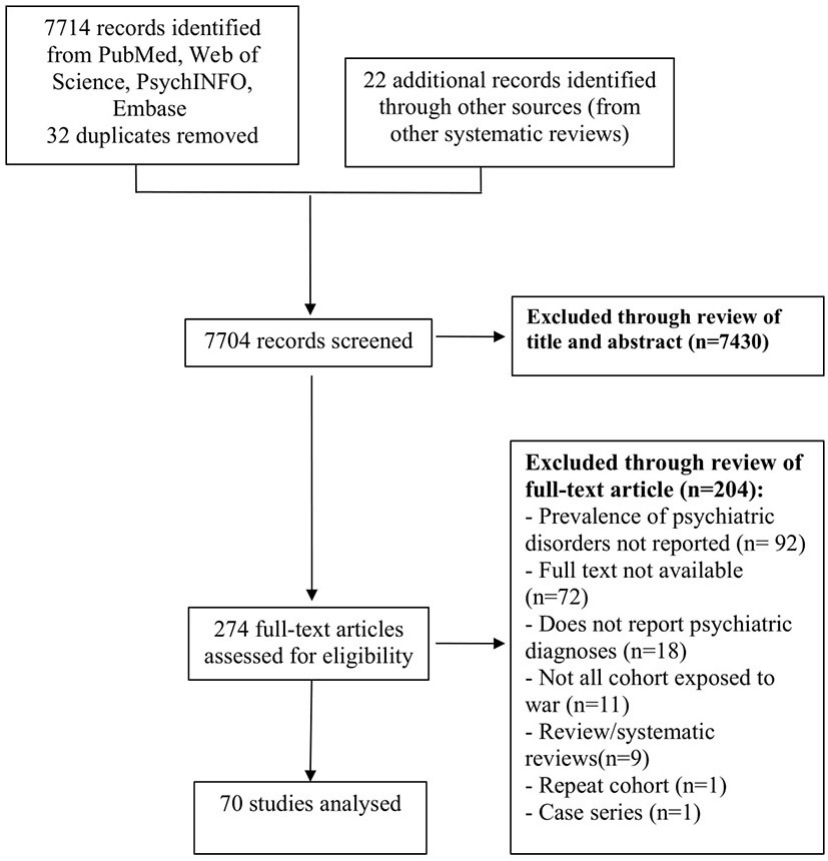
PRISMA flowchart detailing search strategy, inclusion and exclusion criteria.

The preferred reporting items for systematic reviews and meta-analyses (PRISMA) flowchart is presented in Figure 1 (26). The literature search from MEDLINE (Pubmed) and other electronic databases retrieved 7,714 results with 32 duplicates, and bibliographic searches from review articles references uncovered 22 additional studies. After an initial screen, 7,430 studies were excluded through the review of the title and the abstract. In the remaining 274 papers, full text manuscript review and application of inclusion criteria excluded 204 papers, yielding 70 papers to be included in this meta- analysis. The 70 included studies had publication dates ranging from 1982 to 2021. Fifty-seven studies reported on the prevalence of depression, forty-one studies reported on the prevalence of anxiety, and forty-five studies reported on the prevalence of post-traumatic stress.

### The aggregate prevalence of depression

**Figure 2:**
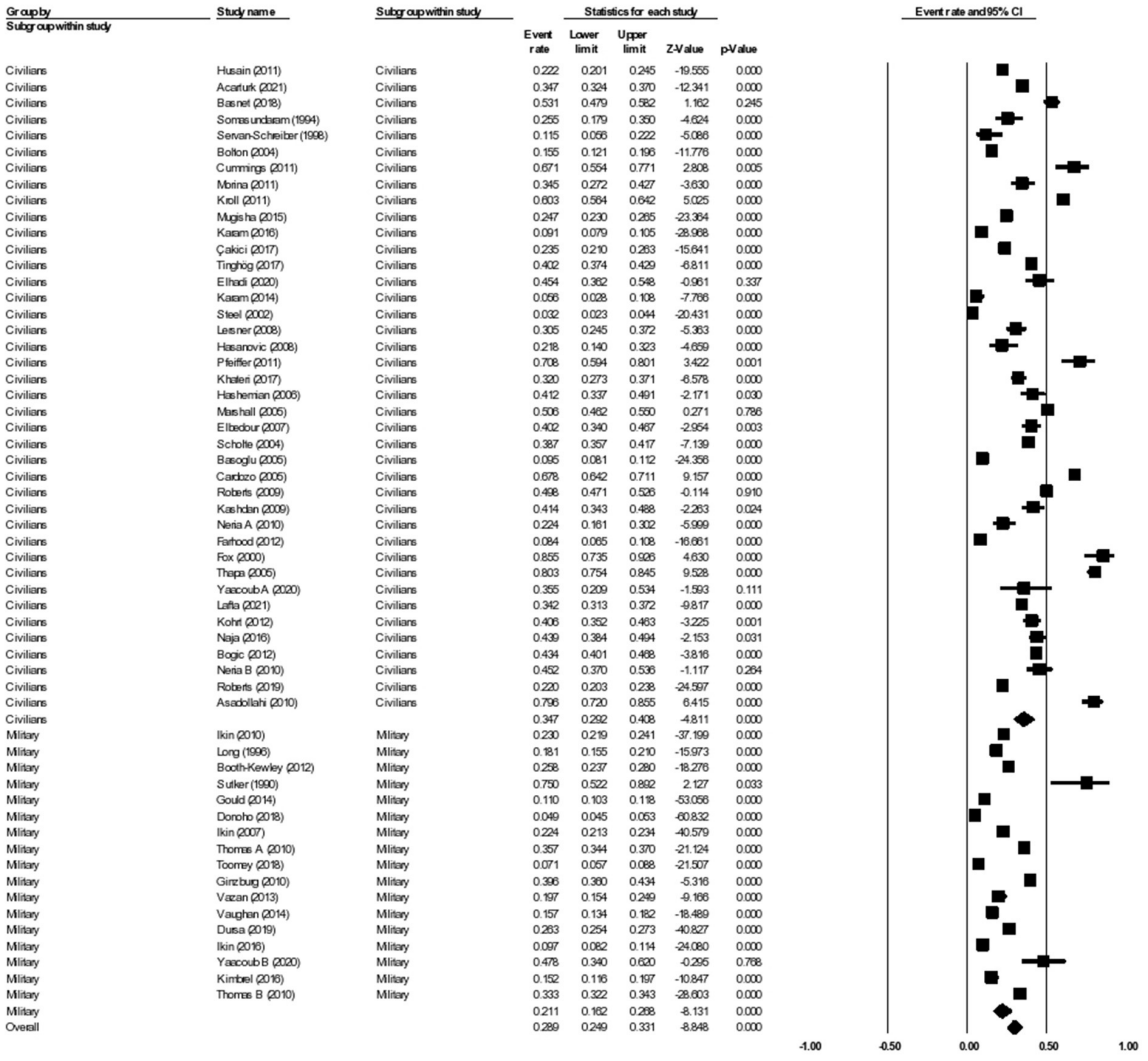
Forest plot showing prevalence of depression, including analysis between military and civilian subgroups.

The 57 studies reporting on the prevalence of depression had a total number of 80,130 participants from 29 different wars or conflicts. Forty studies reported the prevalence of depression among civilians, whereas 17 studies reported on the prevalence among the military. The most common tool used to assess depression was the PHQ-8 or PHQ-9. In the included studies, the prevalence of depression ranged by 3.2–79.6% (27, 28). Overall, in the random-effects model, the aggregate prevalence of depression was 28.9% (95% CI: 23.9–32.1, *I*^2^ = 99.2%) (Figure 2). A linear regression Egger's test of funnel plot asymmetry gave a *p*-value of 0.856, indicating no evidence of publication bias.

#### Subgroup analysis civilian vs military: Depression

When further divided into civilian and military subgroups, the aggregate prevalence of depression among civilians was 33.3% (95% CI: 32.7–34.0, *I*^2^ = 98.6%), while the aggregate prevalence of depression among military personnel was 24.0% (95% CI: 23.6–24.3, *I*^2^ = 99.5%). The subgroup analysis showed significant difference between the two groups, *p* < 0.001. Meta-regression analysis showed that the mean age (Q = 0.01, df = 1, *p* = 0.914) and the proportion of females (Q = 0.118, df = 1, *p* = 0.811) were statistically insignificant and did not explain the heterogeneity observed in the studies.

#### Subgroup analysis during vs post-war: Depression

**Figure 3:**
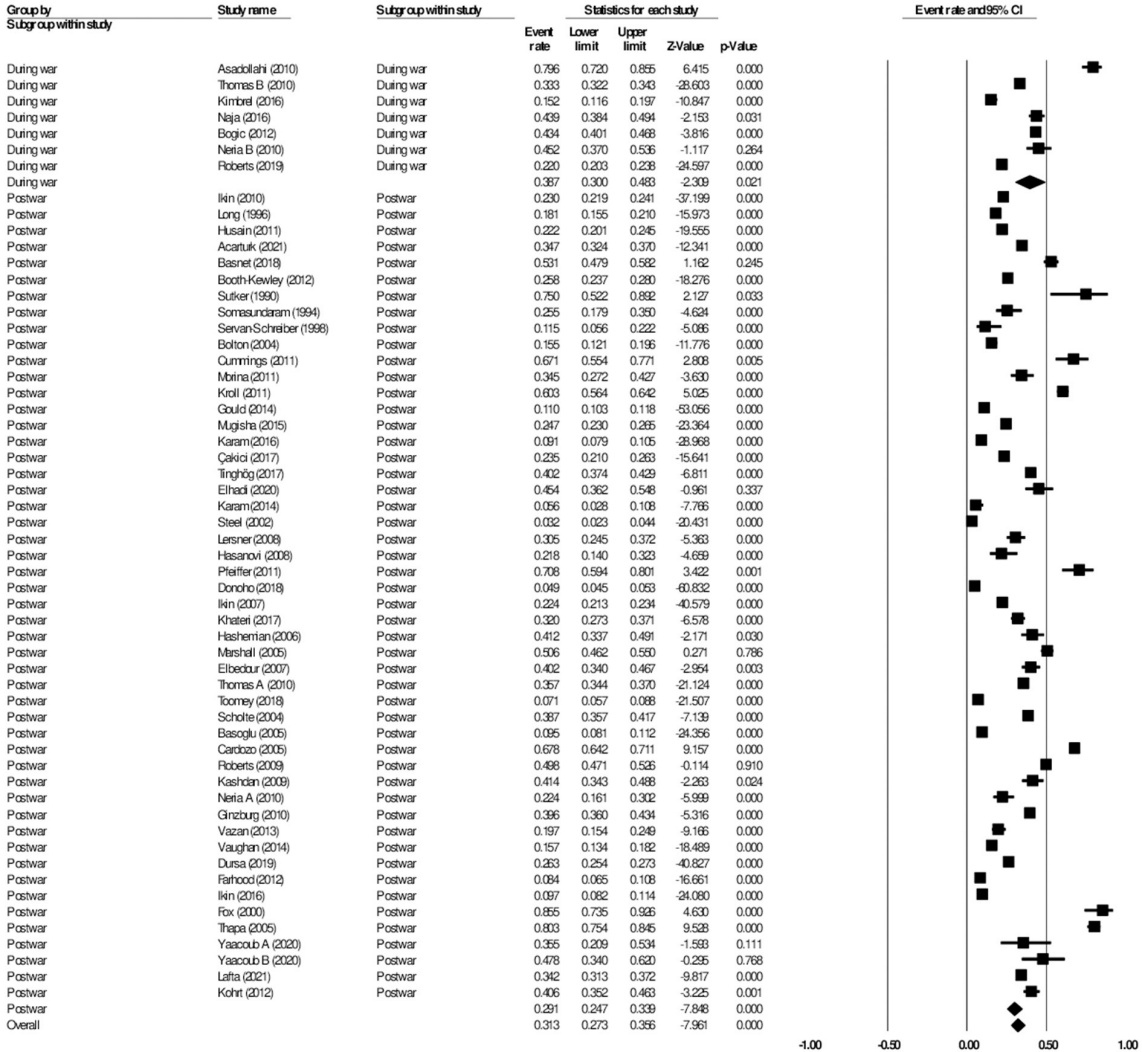
Forest plot showing prevalence of depression during and post-war.

The 57 studies on prevalence of depression were further divided into those that reported on the prevalence of depression during wars or conflicts, and those that reported on the prevalence of depression after the wars or conflicts had ended. There were seven studies that qualified as being reported during the war, while the remaining 44 studies were reported post-war. There was a total of 11,552 participants analyzed during a given war, and 68,578 participants analyzed post-war. The subgroup analysis showed the aggregate prevalence of depression during the wars was 38.7% (95% CI: 30.0–48.3, *I*^2^ = 98.1%), while the aggregate prevalence of depression post-war was 29.1% (95% CI: 24.7–33.9, *I*^2^ = 99.2%). The subgroup analysis showed significant difference between the two groups (*p* < 0.001) (Figure 3).

#### The aggregate prevalence of anxiety

A total of 41 studies were included in the analysis of anxiety, with a total number of 36,948 participants from 22 different wars or conflicts. 31 studies reported the prevalence of anxiety among civilians, whereas 10 studies reported on the prevalence of anxiety among the military. In the included studies, the prevalence of anxiety ranged by 4.2–94.8% (29, 30). Overall, in the random effects model, the prevalence of anxiety was 30.7% (95% CI: 25.5–36.6, *I*^2^ = 99.2%). A linear regression Egger's test of funnel plot asymmetry gave a *p*-value of 0.150, indicating no evidence of publication bias.

#### Subgroup analysis civilian vs military: Anxiety

**Figure 4:**
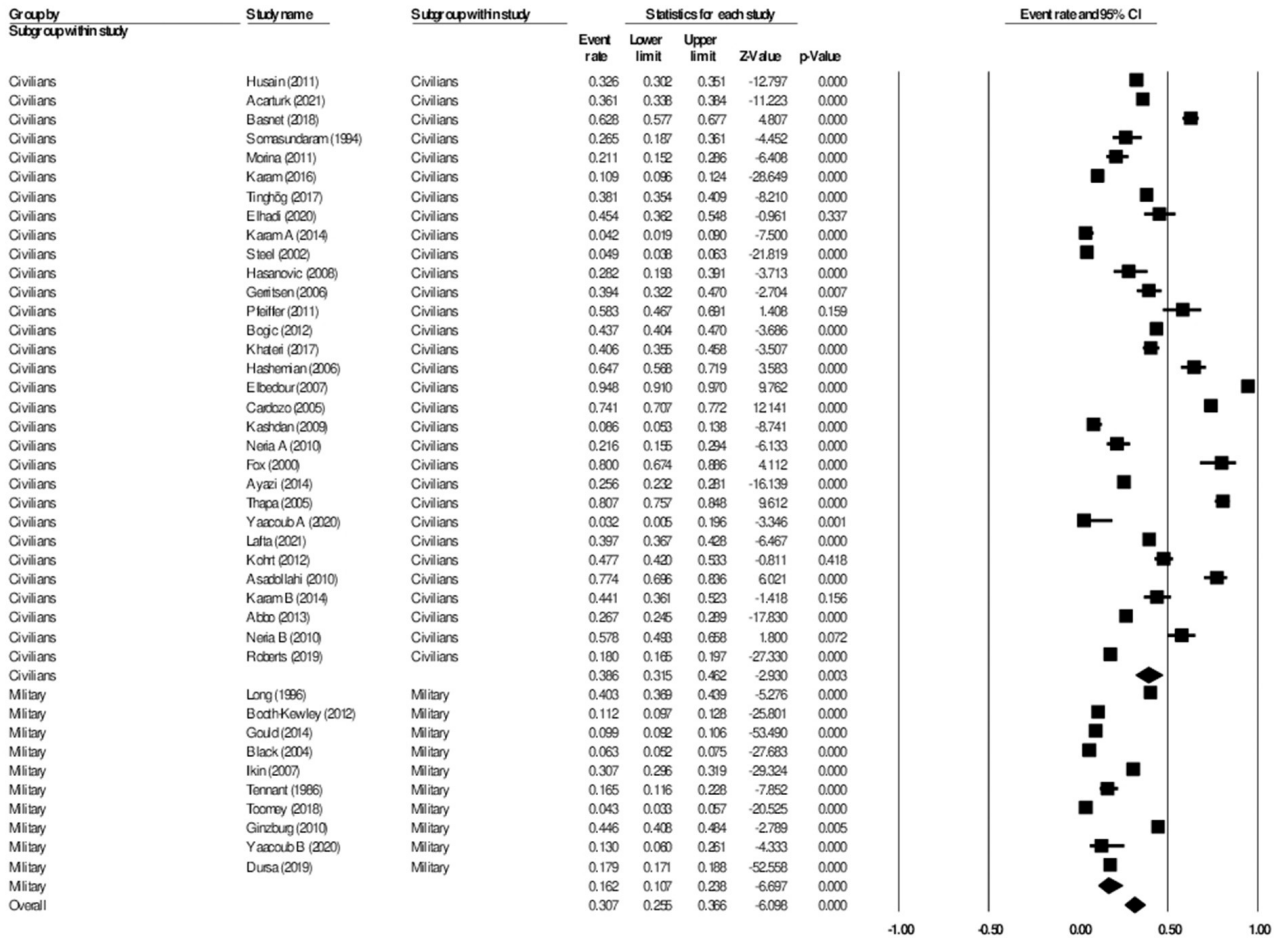
Forest plot showing prevalence of anxiety, including analysis between military and civilian subgroups.

When further divided into civilian and military subgroups, the prevalence of anxiety among civilians was 38.6% (95% CI: 31.5–46.2, *I*^2^ = 98.7%), while the prevalence of anxiety among military personnel was 16.2% (95% CI: 10.7–23.8, *I*^2^ = 99.5%) (see Figure 4). The subgroup analysis yielded significant results between the two groups, *p* < 0.001. Meta-regression analysis showed that the mean age (Q = 0.84, df = 1, *p* = 0.358) and the proportion of females (Q = 0.17, df = 1, *p* = 0.681) were insignificant and did not explain the heterogeneity observed in the studies.

#### Subgroup analysis during vs post-war: Anxiety

The 41 studies on anxiety were divided into those that reported on the prevalence of anxiety during wars or conflicts, and those that reported on the prevalence of anxiety after the wars or conflicts had ended. Five studies qualified as being reported during the war, while the remaining 36 studies were reported post-war. There was a total of 4,205 participants analyzed during a given war, and 32,743 participants analyzed post-war.

**Figure 5:**
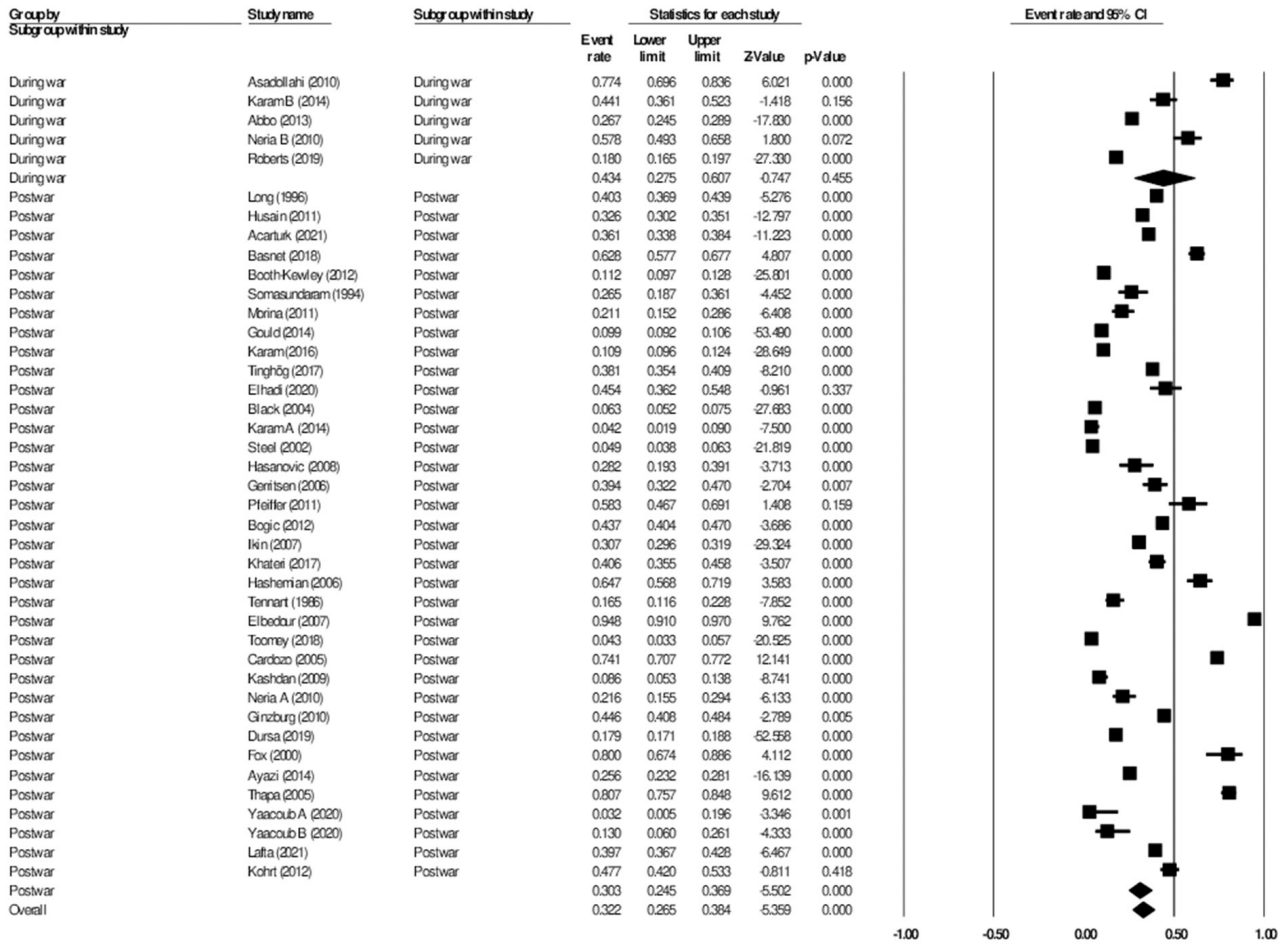
Forest plot showing prevalence of anxiety, including analysis between during war and post-war subgroups.

The subgroup analysis showed the prevalence of anxiety during the wars was 43.4% (95% CI: 27.5–60.7, *I*^2^ = 98.6%), while the prevalence of anxiety post-wars was 30.3% (95% CI: 24.5–36.9, *I*^2^ = 99.2%). The subgroup analysis showed significant difference between the two groups (*p* < 0.001) (Figure 5). Meta-regression analysis showed that the mean age (Q = 0.60, df = 1, *p* = 0.438) and the proportion of females (Q = 0.39, df = 1, *p* = 0.535) were not statistically significant and did not explain the heterogeneity observed in the studies.

### The aggregate prevalence of post-traumatic stress

For studies on post-traumatic stress, a total of 45 studies were included in the analysis, with a total number of 67,153 participants from 23 different wars or conflicts. 31 studies reported the prevalence of post-traumatic stress among civilians, whereas 14 studies reported on the prevalence among the military. In the included studies, the prevalence of post-traumatic stress ranged by 3.9–69.0% (30, 31). Overall, in the random effects model, the prevalence of post-traumatic stress was 23.5% (95% CI: 19.9–27.5, *I*^2^ = 99.0%). A linear regression Egger's test of funnel plot asymmetry gave a *p*-value of 0.988, indicating no evidence of publication bias.

#### Subgroup analysis civilian vs military: Post-traumatic stress

**Figure 6:**
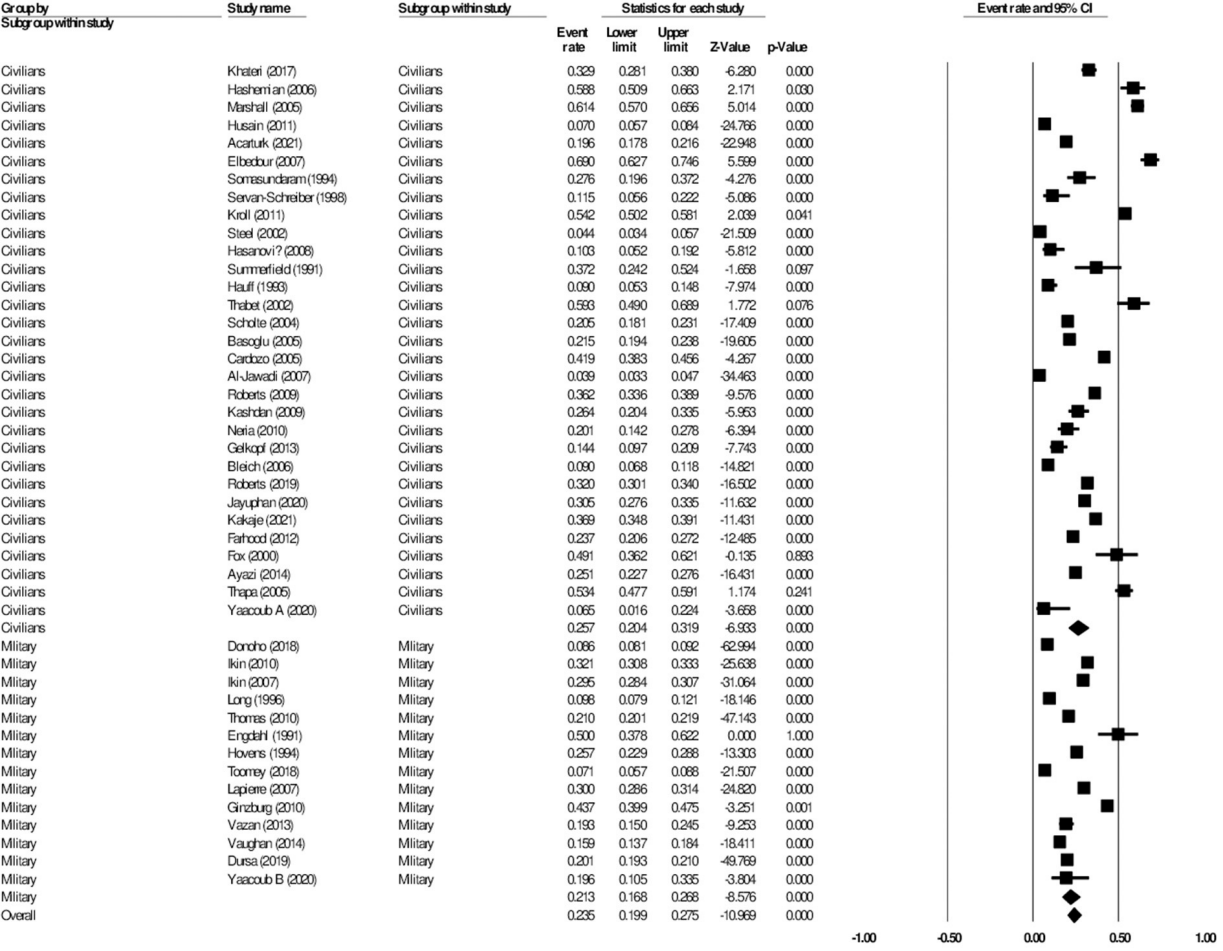
Forest plot showing prevalence of post-traumatic stress disorder, including analysis between military and civilian subgroups.

When further divided into civilian and military subgroups, the prevalence of post-traumatic stress among civilians was 25.7% (95% CI: 20.4–31.9, *I*^2^ = 98.7%), while the prevalence of post-traumatic stress among military personnel was 21.3% (95% CI: 16.8–26.8, *I*^2^ = 99.3%). The subgroup analysis did not show significant difference between the two groups, *p* = 0.256 (Figure 6). Meta-regression analysis showed that the mean age (Q = 0.10, df = 1, *p* = 0.751) and the proportion of females (Q = 0.93, df = 1, *p* = 0.335) did not explain the heterogeneity observed in the studies.

## Discussion

The aggregate prevalence of depression, anxiety and post-traumatic stress during FCV are considerably high, with a prevalence rate of 28.9, 30.7, and 23.5%, respectively. We found significant difference in the levels of depression and anxiety, but not post-traumatic stress between the civilian group and the military group. The aforementioned prevalence is higher than aggregate prevalence of depression among migrants (15.6%) and aggregate lifetime prevalence of depression in the communities (10.8%) (32, 33). The subgroup analysis showed significant difference in prevalence of depression (38.7 vs 29.1%, *p* < 0.001), and anxiety (43.4 vs 30.3%, *p* < 0.001) when comparing during the wars and post-wars, respectively. Controlling for age and gender did not yield significant results in all models tested. To our knowledge, this is the first meta-analysis study to compare the prevalence of psychiatric symptoms between the military and civilian subgroups.

Our study revealed a significant difference in the levels of depression and anxiety between the civilian and the military groups. Interestingly, despite the expected increased level of exposure to violence, combat and threat to life, previous studies have reported that the military group consistently maintained a lower prevalence of psychiatric symptoms (34). For example, studies on the US army report a relatively lower prevalence of mental health symptoms, despite their greater involvement in conflict-afflicted zones (35–37). A possible theory to explain the foregoing observation is that the repeated exposure to violence from military training or previous deployments have desensitized the military troops, evoking a less drastic emotional response compared to that of the general population (38). However, an exception to the foregoing theory would be the population turned prisoners-of-war in Sutker et al. (39), which reported significantly higher prevalence rate of depression and anxiety (75 and 45%, respectively), owing to their biological and psychological abuse including but not limited to poorer living conditions, separation from home country and torture. In general, despite the lower prevalence in the military subgroup, our results must be interpreted with extreme caution for it is not a justification for less resources directed to the mental health of the military group, but rather emphasizes the need for universal and accessible mental wellness resources to the affected population, including humanitarian interventions to enhance cultural and social identity toward the war-weakened survivor populations (40). Another group whose reactions could be the subject of further meta-analyses are professionals involved in assisting victims of war and refugees. It would be important to identify the determinants of the reactions of people providing support, like medical staff members. Their psychological reaction and mental health condition, although they have only indirect contact with war, through victims' observations, can be another important source of knowledge about the effects of war on human psychological condition (41).

Due to the expected long-term involvement in conflict- or war-afflicted zones, and time away from their home country, the US military ensures that they are well-equipped with mental health tools and resources to aid their troops. Mental Health Care in the US troops is seen with utmost importance, integrating mental care into their daily work life *via* centralized workload management; consolidation of professionally trained mental health domains under integrated behavioral health departments such as psychiatry, clinical psychology, psychiatric nursing, and social work services; creation of satellite mental health clinics embedded within brigade work areas; extension of psychosocial help to also the soldier's family members to name a few (42). From our analyses, the US army reports much lower prevalence of psychiatric symptoms (7.10–33.3%) (43, 44), compared to their Israelian counterparts (39.6–47.8%) (45, 46), suggesting that integration of mental health resources is beneficial to the mental wellness of a population (47). While not explored in this paper, we recommend that further research can be done to investigate if there is the presence of non-report bias, or other stigma associated with reduced report of mental health symptoms to improve the detection and outreach of at-risk individuals in the military. There was no significant difference between prevalence of post-traumatic stress in the civilian population and military population in this meta-analysis. We recommend a thorough analysis on the burden of diseases, and the risk factors associated with post-traumatic stress to identify vulnerable groups, and to better inform aid and resources for at-risk individuals.

When comparing the prevalence of depression and anxiety during vs post-war, both showed a significant downward trend after a given war had ended from 38.7 to 26.2% and from 43.4 to 28.1%, respectively. Significant contributing factors to the observed trend include interventions such as perceived social support and immediate emotional support to affected individuals using comprehensive trauma-informed response to provide appropriate mental health care (48, 49). The results suggest that the removal of the perpetuating stress factor of war can significantly reduce the morbidity of the general population, and that for a significant portion of the affected population, the morbidity is often not long-lasting (5, 27, 49–52). In addition to removing the traumatic stimuli, adequate financial and mental health aid from the government and a stable political climate can reduce the long-term morbidity associated with increased FCV (27, 48, 53). Factors that were associated with increased prevalence of depression and anxiety included a lower level of education, more traumatic experiences during the times of conflict, migratory stresses, alcohol misuse and known aggressive behavior (5, 44). One study found that increased conflict-exposure was more likely associated with increased rates of anxiety, whereas socioeconomic factors played a greater role as a predictor of depression (53). Further research is required to identify moderational factors of the effect of FCV on psychiatric symptoms of persons living in war-afflicted areas.

### Strengths and limitations

The study herein has multiple strengths to be considered. Firstly, our study assessed multiple populations that may be affected by war, including and not limited to civilians, military, prisoners-of-war and refugees. Our subgroup analysis focused on civilians and the military, which highlighted how the availability of mental health resources influences the susceptibility of a population to mental disorders. It also evaluated the temporal relationship of depression and war, and how war acts as a perpetuating factor for depression but can decrease significantly once the stimuli (i.e., war) is removed (54). Studies included were also conducted in a broad range of diverse countries, which may reduce potential biases with respect to cultural or ethnic differences (i.e., potentially increases the generalizability of our results). Moreover, all results reported did not have publication bias.

However, there are multiple limitations that should be considered when interpreting the results. Firstly, our meta-analysis had a high level of heterogeneity. However, it should also be considered that high heterogeneity in a meta-analysis may be expected when analyzing a large number of studies. Furthermore, there are great differences in the evaluation of depression, anxiety and post-traumatic stress due to the different tools and questionnaires that are used—differences in assessment tools may also affect the corresponding reported prevalence. Secondly, meta-regression was only performed on selected demographics such as age and gender as there was limited data available on other factors including level of education, married or divorce status, exposure to trauma. Thirdly, due to limitations to time and resources, not all databases such as Scopus, a trustable database, was included in the analysis, which may have left out important studies in the analysis. Fourthly, this meta-analysis spanned many years from 1982 to 2021, during which there have been many changes in the diagnostic criteria of mental disorders, thus affecting the accuracy of the values reported and the validity of the results during the time period this paper was written. Lastly, in terms of interpretation of results, meta-regression denotes an observational association and is limited by ecological fallacy (55).

### Practical and clinical implications of study

The aggregate prevalence of depression, anxiety and post-traumatic stress in populations were relatively high in populations experiencing FCV, with a significant difference in prevalence of depression and anxiety between civilians and the military personnels. Surprisingly, the military population had a lower prevalence of mental health symptoms, which literature review may owe to their more comprehensive mental health interventions, or desensitization to war and violence. Our results also show a significant difference in the prevalence of depression and anxiety among individuals in areas affected by FCV during the wars compared to after the wars, with supporting literature suggesting a concurrent decrease in healthcare-seeking behavior during times of conflict.

According to Feldstein, a systematic review of research plays an important role in the process of translating scientific evidence into patient care decisions, allowing the clinical practice to be organized based on scientific evidence from multiple studies and identifying new research topics, thus contributing to the development of science (56).

We believe that our study is an important step toward finding possible solutions, and highlighting key problems during a time where mental health issues are easily overlooked. We believe it carries important practical implications, as it helps identify groups that are vulnerable to mental health risks during and after the war, making it possible to target psychiatric care to people of the studied groups. The results of the study show what are the specific disorders of their members and what is the intensity of the most common symptoms. This knowledge seems important for planning appropriate forms of help.

The lack of significant differences between groups with regard to post-traumatic stress severity dictates that we should look carefully at the treatment provided during and after the war conflicts, conducted for civilians in terms of counteracting the negative effects of PTSD and mitigating those that have occurred. In light of the data obtained, it seems even more important to look for ways to stop or alleviate the suffering of innocent people who are unprepared to take part in combat and are painfully surprised by the participation in traumatic situations, the brutal consequences of which they have to face.

## Conclusion

The prevalence of depression, anxiety and post-traumatic stress in populations with FCV are 28.9, 30.7, and 23.5%, respectively. There is a significant difference in the prevalence of depression and anxiety in civilians and the military troops, but no significant difference between groups with respect to post-traumatic stress. Lastly, there is a significant difference in the prevalence of depression and anxiety during the war compared to post-war. We emphasize interpreting the results carefully and recommend the importance of access to mental health resources, especially for at-risk persons living with FCV.

## Data availability statement

The original contributions presented in the study are included in the article/Supplementary material, further inquiries can be directed to the corresponding author/s.

## Author contributions

RH designed and conceptualized the study. IL was responsible for data collection, data analysis, and drafted the initial manuscript. WT, AC-C, RM, KT, RH, and CH read the article for critical revision. All authors contributed to the article and approved the submitted version.

## Funding

This study was funded by NUS Department of Psychological Medicine (R-177-000-100-001/R-177-000-003-001/ R177000702733) and NUS iHealthtech Other Operating Expenses (R-722-000-004-731).

## Conflict of interest

RM has received research grant support from CIHR/GACD/National Natural Science Foundation of China (NSFC); speaker/consultation fees from Lundbeck, Janssen, Alkermes, Neumora Therapeutics, Boehringer Ingelheim, Sage, Biogen, Mitsubishi Tanabe, Purdue, Pfizer, Otsuka, Takeda, Neurocrine, Sunovion, Bausch Health, Axsome, Novo Nordisk, Kris, Sanofi, Eisai, Intra-Cellular, NewBridge Pharmaceuticals, Abbvie, Atai Life Sciences. RM is a CEO of Braxia Scientific Corp. KT has received personal fees from Braxia Scientific Corp.

## Publisher's note

All claims expressed in this article are solely those of the authors and do not necessarily represent those of their affiliated organizations, or those of the publisher, the editors and the reviewers. Any product that may be evaluated in this article, or claim that may be made by its manufacturer, is not guaranteed or endorsed by the publisher.
