# Supplementary material for: Prevalence of depression, anxiety and post-traumatic stress in war- and conflict-afflicted areas: A meta-analysis
Source: Front Psychiatry. 2022 Sep 16;13:978703. doi: 10.3389/fpsyt.2022.978703 (PMC9524230; doi:10.3389/fpsyt.2022.978703)
Supplement: Supplementary file 1 [file Data_Sheet_1.docx]

Appendix 1: Included Studies

Summary of depression references

| Full reference | Study population | Survey year | Years of war/conflict | Sample size | Mean age | Proportion of females | Tool used | Country (of study) |
| --- | --- | --- | --- | --- | --- | --- | --- | --- |
| Ikin et al (2010)  Comorbidity of PTSD and depression in Korean War veterans: prevalence, predictors, and impairment | Military |  | 1950-1953 | 5352 | 75.0 |  | HADS | Australia |
| Long et al (1996)  Prevalence of posttraumatic stress disorder, depression and anxiety in a community sample of New Zealand Vietnam War veterans | Military |  | 1955-1975 | 756 | 50.0 |  | BDI | New Zealand |
| Husain et al (2011)  Prevalence of war-related mental health conditions and association with displacement status in postwar Jaffna District, Sri Lanka | Civilians | 2009 | 1995 | 1409 | 40.2 | 0.675 | HSC-25 | Sri Lanka |
| Acarturk et al (2021)  Prevalence and predictors of common mental disorders among Syrian refugees in Istanbul, Turkey: a cross-sectional study | Civilians | 2018 | 2011-present | 1678 |  | 0.516 | HSC-25 | Syria |
| Basnet et al (2018)  Depression and anxiety among war-widows of Nepal: a post-civil war cross-sectional study | Civilians | 2012 | 1996-2006 | 358 |  | 1 | BDI | Nepal |
| Booth-Kewley et al (2012)  Anxiety and depression in Marines sent to war in Iraq and Afghanistan | Military | 2008 | Multiple | 1560 |  | 0.051 | CES-D | United States |
| Sutker et al (1990)  Assessment of long-term psychosocial sequelae among POW survivors of the Korean Conflict | Military |  | 1950-1953 | 20 |  |  |  | United States |
| Somasundaram et al (1994)  War trauma in a civilian population | Civilians | 1991 | 1995 | 98 |  | 0.357 | DSM-III-R | Sri Lanka |
| Servan-Schreiber et al (1998)  Prevalence of posttraumatic stress disorder and major depressive disorder in Tibetan refugee children | Civilians | 1997 | 1951 | 61 |  | 0.508 | DSM-IV-R | Tibet |
| Bolton et al (2004)  Prevalence of depression in rural Rwanda based on symptom and functional criteria | Civilians | 1999 | 1994 | 368 | 35.7 | 0.660 | HSC-25 | Rwanda |
| Cummings et al (2011)  Correlates of depression among older Kurdish refugees | Civilians |  | 1978-present | 70 | 59.0 | 0.529 | GDS | United States |
| Morina et al (2011)  Long-term outcomes of war-related death of family members in Kosovar civilian war survivors | Civilians | 2008 | 1998-1999 | 142 | 38.2 | 0.662 | MINI | Kosovo |
| Kroll et al (2011)  Psychoses, PTSD, and depression in Somali refugees in Minnesota | Civilians | 2009 | 1991-present | 600 |  | 0.535 | DSM-IV-R | United States |
| Gould et al (2014)  Depression and anxiety symptoms in male veterans and non-veterans: the Health and Retirement Study | Military | 2006 | Multiple | 6577 |  |  | CES-D | United States |
| Mugisha et al (2015)  Major depressive disorder seven years after the conflict in northern Uganda: burden, risk factors and impact on outcomes (The Wayo-Nero Study) | Civilians | 2013 | 1986-2006 | 2361 |  | 0.625 | MINI | Uganda |
| Karam et al (2016)  Prevalence, Correlates, and Treatment of Mental Disorders among Lebanese Older Adults: A National Study | Civilians |  | 1975-1990 | 1915 |  | 0.493 | DSM-IV-R | Lebanon |
| Çakıcı et al (2017)  Depression: point-prevalence and risk factors in a North Cyprus household adult cross-sectional study | Civilians | 2016 | 1974 | 978 |  | 0.474 | BDI | Cyprus |
| Tinghög et al (2017)  Prevalence of mental ill health, traumas and postmigration stress among refugees from Syria resettled in Sweden after 2011: a population-based survey | Civilians | 2016 | 2011-present | 1215 |  | 0.372 | HSC-25 | Sweden |
| Elhadi et al (2020)  Prevalence of anxiety and depressive symptoms among emergency physicians in Libya after civil war: a cross-sectional study | Civilians | 2018 | 2014-2020 | 108 | 31.2 | 0.315 | HADS | Libya |
| Karam et al (2014)  Outcome of depression and anxiety after war: a prospective epidemiologic study of children and adolescents | Civilians | 1997 | 1975-1990 | 143 | 11.5 | 0.500 | DICA-R | Lebanon |
| Steel et al (2002)  Long-term effect of psychological trauma on the mental health of Vietnamese refugees resettled in Australia: a population-based study | Civilians |  | 1955-1975 | 1161 | 41.0 | 0.590 | ICD-10 | Australia |
| Lersner et al (2008)  Mental health of returnees: refugees in Germany prior to their state-sponsored repatriation | Civilians | 2005 | Multiple | 200 | 43.2 | 0.500 | MINI | Germany |
| Hasanović et al (2008)  Post traumatic stress disorder, depression and anxiety among family medicine residents after 1992-95 war in Bosnia and Herzegovina | Civilians | 2004 | 1992-1995 | 78 |  | 0.846 | HSC-25 | Bosnia and Herzegovina |
| Pfeiffer et al (2011)  PTSD, depression and anxiety among former abductees in Northern Uganda | Civilians | 2005 | 1986-2006 | 72 | 23.7 |  | HSC-25 | Uganda |
| Donoho et al (2018)  Depression among military spouses: Demographic, military, and service member psychological health risk factors | Civilians | 2011 | Multiple | 9038 |  | 0.87 | PHQ-9 | United States |
| Ikin et al (2007)  Anxiety, post-traumatic stress disorder and depression in Korean War veterans 50 years after the war | Military | 2005 | 1950-1953 | 6122 | 75 |  | HADS | Australia |
| Khateri et al (2017)  Mental health status following severe sulfur mustard exposure: a long-term study of Iranian war survivors | Civilians |  | 1980-1988 | 350 | 45.2 |  | DSM-IV-R | Iran |
| Hashemian et al (2006)  Anxiety, depression, and posttraumatic stress in Iranian survivors of chemical warfare | Civilians | 2004 | 1980-1988 | 153 | 45 |  | BDI | Iran |
| Marshall et al (2005)  Mental Health of Cambodian Refugees 2 Decades After Resettlement in the United States | Civilians | 2005 | 1955-1975, 1978-1989 | 490 | 52 | 0.61 | CIDI | United States |
| Elbedour et al (2007)  Post-traumatic stress disorder, depression, and anxiety among Gaza Strip adolescents in the wake of the second Uprising (Intifada) | Civilians |  | 2000-2005 | 229 | 17.1 | 0.472 | BDI | Gaza Strip |
| Thomas et al (2010)  Prevalence of mental health problems and functional impairment among active component and National Guard soldiers 3 and 12 months following combat in Iraq | Military | 2004 | Multiple | 5609 |  | 0.039 | PHQ-9 | United States |
| Toomey et al (2018)  Mental health of US Gulf War veterans 10 years after the war | Military | 1991 | 1990-1991 | 1061 | 38.9 | 0.22 | BDI | United States |
| Scholte et al (2004)  Mental health symptoms following war and repression in eastern Afghanistan | Civilians | 2003 | 1979-1989 | 1011 |  | 0.55 | HSC-25 | Afghanistan |
| Başoglu et al (2005)  Psychiatric and cognitive effects of war in former Yugoslavia: association of lack of redress for trauma and posttraumatic stress reactions | Civilians | 2000 | 1991-2001 | 1358 | 39 | 0.41 | DSM-IV-R | Yugoslavia |
| Cardozo et al (2005)  Report from the CDC: mental health of women in postwar Afghanistan | Civilians | 2002 | 2001-2014 | 695 |  | 0.609 | HSC-25 | Afghanistan |
| Roberts et al (2009)  Post-conflict mental health needs: a cross-sectional survey of trauma, depression and associated factors in Juba, Southern Sudan | Civilians | 2007 | 1983-2005 | 1242 | 33 | 0.507 | HSC-25 | Sudan |
| Kashdan et al (2009)  Post-traumatic stress disorder, social anxiety disorder, and depression in survivors of the Kosovo War: experiential avoidance as a contributor to distress and quality of life | Civilians | 2006 | 1998-1999 | 164 |  |  | MINI | Kosovo |
| Neria et al (2010)  A longitudinal study of posttraumatic stress disorder, depression, and generalized anxiety disorder in Israeli civilians exposed to war trauma | Civilians | 2009 | 2008-2009 | 134 | 24 | 0.84 | PHQ-9 | Israel |
| Ginzburg et al (2010)  Comorbidity of posttraumatic stress disorder, anxiety and depression: a 20-year longitudinal study of war veterans | Military | 1983 | Multiple | 664 |  |  | DASSC-90 | Israel |
| Vazan et al (2013) Substance use and other mental health disorders among veterans returning to the inner city: prevalence, correlates, and rates of unmet treatment need | Military | 2011 | Multiple | 269 |  | 0.122 | PHQ-9 | United States |
| Vaughan et al (2014)  Prevalence of mental health problems among Iraq and Afghanistan veterans who have and have not received VA services | Military | 2010 | Multiple | 913 |  | 0.11 | PHQ-8 | United States |
| Dursa et al (2019)  Health Status of Female and Male Gulf War and Gulf Era Veterans: A Population-Based Study | Military | 2012 | 1990-1991 | 8104 |  | 0.194 | PHQ-15 | United States |
| Farhood et al (2012)  Prevalence and predictors for post-traumatic stress disorder, depression and general health in a population from six villages in South Lebanon | Civilians | 2009 | 1985-2000 | 632 |  |  | BDI | Lebanon |
| Ikin et al (2016)  Major depression and depressive symptoms in Australian Gulf War veterans 20 years after the Gulf War | Military | 1990 | 1990-1991 | 1330 | 49.4 | 0.020 | PHQ-9 | United States |
| Fox et al (2000) The Sierra Leonean refugee experience: traumatic events and psychiatric sequelae | Civilians |  | 1991-2002 | 55 | 31.3 | 0.491 | HSC-25 | West Africa |
| Thapa et al (2005) Psychological distress among displaced persons during an armed conflict in Nepal | Civilians | 2003 | 1996-2006 | 290 | 40.9 | 0.390 | HSC-25 | Nepal |
| Yaacoub et al A (2020) Posttraumatic stress disorders and depression among male inpatient adults involved in the Lebanese war | Civilians | 2016 | 2016 | 31 | 42.6 | 0 | HADS and MINI | Lebanon |
| Yaacoub et al B (2020) Posttraumatic stress disorders and depression among male inpatient adults involved in the Lebanese war | Military | 2016 | 2016 | 31 | 42.6 | 0 | HADS and MINI | Lebanon |
| Lafta et al (2021) Women's mental health in Iraq post-conflict | Civilians | 2020 | 2003 | 1000 |  | 1 | DSM-V | Iran |
| Kohrt et al (2012) Political violence and mental health in Nepal: prospective study | Civilians | 2007 | 2000-2007 | 298 |  | 0.436 | BDI | Nepal |
| Asadollahi et al (2010)  Anxiety, depression and health-related quality of life in those injured by landmines, Ilam, Islamic Republic of Iran | Civilians | 2007 | 1980-1988 | 137 | 38.1 | 0.066 | BDI | Iran |
| Kimbrel et al (2016)  A 12-Month prospective study of the effects of PTSD-depression comorbidity on suicidal behavior in Iraq/Afghanistan-era veterans | Military |  | Multiple | 309 | 38.8 | 0.324 |  | United States |
| Naja et al (2016)  Prevalence of depression in Syrian refugees and the influence of religiosity | Civilians | 2014 | 2011-present | 310 |  | 0.612 | MINI | Lebanon |
| Bogic et al (2012)  Factors associated with mental disorders in long-settled war refugees: refugees from the former Yugoslavia in Germany, Italy and the UK | Civilians | 2005 | 1991-2001 | 841 |  | 0.513 | MINI | Germany, Italy, United Kingdom |
| Roberts et al (2019) Mental health care utilisation among internally displaced persons in Ukraine: results from a nation-wide survey | Civilians | 2016 | 2014 | 2203 |  | 0.681 | PHQ-9 | Ukraine |

Abbreviations: Hospital Anxiety and Depression Scale – HADS; Beck Depression Inventory – BDI; Hopkins Symptom Checklist-25 – HSC-25; Center for Epidemiologic Studies Depression Scale – CES-D; Diagnostic and Statistical Manual of Mental Disorders III-Revised – DSM-III-R; Diagnostic and Statistical Manual of Mental Disorders IV-Revised – DSM-IV-R; Geriatric Depression Scale – GDS; Mini International Neuropsychiatric Interview – MINI; Diagnostic Interview for Children and Adolescents Revised – DICA-R; International Classification of Diseases-10 – ICD-10; Patient Health Questionnaire – PHQ; Composite International Diagnostic Interview – CIDI; Depression and Anxiety Subscales of the Symptoms Checklist-90 – DASSC-90;

Summary of anxiety references

| Full reference | Study population | Survey year | Years of war/conflict | Sample size | Mean age | Proportion of females | Tool used | Country |
| --- | --- | --- | --- | --- | --- | --- | --- | --- |
| Long et al (1996)  Prevalence of posttraumatic stress disorder, depression and anxiety in a community sample of New Zealand Vietnam War veterans | Military |  | 1955-1975 | 756 | 50.0 |  | STAI | New Zealand |
| Husain et al (2011)  Prevalence of war-related mental health conditions and association with displacement status in postwar Jaffna District, Sri Lanka | Civilians | 2009 | 1995 | 1409 | 40.2 | 0.675 | HSC-25 | Sri Lanka |
| Acarturk et al (2021)  Prevalence and predictors of common mental disorders among Syrian refugees in Istanbul, Turkey: a cross-sectional study | Civilians | 2018 | 2011-present | 1678 |  | 0.516 | HSC-25 | Syria |
| Basnet et al (2018)  Depression and anxiety among war-widows of Nepal: a post-civil war cross-sectional study | Civilians | 2012 | 2000-2007 | 358 |  | 1 | BAI | Nepal |
| Booth-Kewley et al (2012)  Anxiety and depression in Marines sent to war in Iraq and Afghanistan | Military | 2008 | Multiple | 1560 |  | 0.051 | BAI | United States |
| Somasundaram et al (1994)  War trauma in a civilian population | Civilians | 1991 | 1995 | 98 |  | 0.357 | DSM-III-R | Sri Lanka |
| Morina et al (2011)  Long-term outcomes of war-related death of family members in Kosovar civilian war survivors | Civilians | 2008 | 1998-1999 | 142 | 38.2 | 0.662 | DSM-IV-R | Kosovo |
| Gould et al (2014)  Depression and anxiety symptoms in male veterans and non-veterans: the Health and Retirement Study | Military | 2006 | Multiple | 6577 |  |  | BAI | United States |
| Karam et al (2016)  Prevalence, Correlates, and Treatment of Mental Disorders among Lebanese Older Adults: A National Study | Civilians |  | 1975-1990 | 1915 |  | 0.493 | DSM-IV-R | Lebanon |
| Tinghög et al (2017)  Prevalence of mental ill health, traumas and postmigration stress among refugees from Syria resettled in Sweden after 2011: a population-based survey | Civilians | 2016 | 2011-present | 1215 |  | 0.372 | HSC-25 | Sweden |
| Elhadi et al (2020)  Prevalence of anxiety and depressive symptoms among emergency physicians in Libya after civil war: a cross-sectional study | Civilians | 2018 | 2014-2020 | 108 | 31.2 | 0.315 | HADS | Libya |
| Black et al (2004)  Gulf War veterans with anxiety: prevalence, comorbidity, and risk factors | Military | 1995 | 1990-1991 | 1782 |  |  | DSM-IV-R | United States |
| Karam et al (2014)  Outcome of depression and anxiety after war: a prospective epidemiologic study of children and adolescents | Civilians | 1997 | 1975-1990 | 143 | 11.5 | 0.500 | DICA-R | Lebanon |
| Steel et al (2002)  Long-term effect of psychological trauma on the mental health of Vietnamese refugees resettled in Australia: a population-based study | Civilians |  | 1955-1975 | 1161 | 41.0 | 0.590 | ICD-10 | Australia |
| Hasanović et al (2008)  Post traumatic stress disorder, depression and anxiety among family medicine residents after 1992-95 war in Bosnia and Herzegovina | Civilians | 2004 | 1992-1995 | 78 |  | 0.846 | HSC-25 | Bosnia and Herzegovina |
| Gerritsen et al (2006)  Physical and mental health of Afghan, Iranian and Somali asylum seekers and refugees living in the Netherlands | Civilians | 2003 | 1980-1988 | 165 | 40.3 | 0.444 | HSC-25 | Netherlands |
| Pfeiffer et al (2011)  PTSD, depression and anxiety among former abductees in Northern Uganda | Civilians | 2005 | 1986-2006 | 72 | 23.7 |  | HSC-25 | Uganda |
| Bogic et al (2012)  Factors associated with mental disorders in long-settled war refugees: refugees from the former Yugoslavia in Germany, Italy and the UK | Civilians | 2005 | 1991-2001 | 841 |  | 0.513 | MINI | Germany, Italy, United Kingdom |
| Ikin et al (2007)  Anxiety, post-traumatic stress disorder and depression in Korean War veterans 50 years after the war | Military | 2005 | 1950-1953 | 6122 | 75 |  | HADS | Australia |
| Khateri et al (2017)  Mental health status following severe sulfur mustard exposure: a long-term study of Iranian war survivors | Civilians |  | 1980-1988 | 350 | 45.2 |  | DSM-IV-R | Iran |
| Hashemian et al (2006)  Anxiety, depression, and posttraumatic stress in Iranian survivors of chemical warfare | Civilians | 2004 | 1980-1988 | 153 | 45 |  | HSA | Iran |
| Tennant et al (1986)  Australian prisoners of war of the Japanese: post-war psychiatric hospitalisation and psychological morbidity | Military |  | 1937-1945 | 170 |  |  | DSM-III-R | Australia |
| Elbedour et al (2007)  Post-traumatic stress disorder, depression, and anxiety among Gaza Strip adolescents in the wake of the second Uprising (Intifada) | Civilians |  | 2000-2005 | 229 | 17.1 | 0.472 | BAI | Gaza Strip |
| Toomey et al (2018)  Mental health of US Gulf War veterans 10 years after the war | Military | 1991 | 1990-1991 | 1061 | 38.9 | 0.22 | BAI | United States |
| Scholte et al (2004)  Mental health symptoms following war and repression in eastern Afghanistan | Civilians | 2003 | 1979-1989 | 1011 |  | 0.55 | HSC-25 | Afghanistan |
| Cardozo et al (2005)  Report from the CDC: mental health of women in postwar Afghanistan | Civilians | 2002 | 1979-1989 | 695 |  | 0.609 | HSC-25 | Afghanistan |
| Kashdan et al (2009)  Post-traumatic stress disorder, social anxiety disorder, and depression in survivors of the Kosovo War: experiential avoidance as a contributor to distress and quality of life | Civilians | 2006 | 1998-1999 | 164 |  |  | MINI | Kosovo |
| Neria et al (2010)  A longitudinal study of posttraumatic stress disorder, depression, and generalized anxiety disorder in Israeli civilians exposed to war trauma | Civilians | 2009 | 2008-2009 | 134 | 24 | 0.84 | GAD-7 | Israel |
| Ginzburg et al (2010)  Comorbidity of posttraumatic stress disorder, anxiety and depression: a 20-year longitudinal study of war veterans | Military | 1983 | Multiple | 664 |  |  | DASSC-90 | Israel |
| Dursa et al (2019)  Health Status of Female and Male Gulf War and Gulf Era Veterans: A Population-Based Study | Military | 2012 | 1990-1991 | 8104 |  | 0.194 | PHQ-15 | United States |
| Fox et al (2000)  The Sierra Leonean refugee experience: traumatic events and psychiatric sequelae | Civilians |  | 1991-2002 | 55 | 31.3 | 0.491 | HSC-25 | West Africa |
| Ayazi et al (2014) Association between exposure to traumatic events and anxiety disorders in a post-conflict setting: a cross-sectional community study in South Sudan | Civilians |  | 1983-2005 | 1200 |  | 0.436 | MINI | South Sudan |
| Thapa et al (2005) Psychological distress among displaced persons during an armed conflict in Nepal | Civilians | 2003 | 1996-2006 | 290 | 40.9 | 0.390 | HSC-25 | Nepal |
| Yaacoub et al A (2020) Posttraumatic stress disorders and depression among male inpatient adults involved in the Lebanese war | Civilians | 2016 | 2016 | 31 | 42.6 | 0 | HADS and MINI | Lebanon |
| Yaacoub et al B (2020) Posttraumatic stress disorders and depression among male inpatient adults involved in the Lebanese war | Military | 2016 | 2016 | 31 | 42.6 | 0 | HADS and MINI | Lebanon |
| Lafta et al (2021) Women's mental health in Iraq post-conflict | Civilians | 2020 | 2003 | 1000 |  | 1 | DSM-V | Iran |
| Kohrt et al (2012) Political violence and mental health in Nepal: prospective study | Civilians | 2007 | 2000-2007 | 298 |  | 0.436 | BDI | Nepal |
| Asadollahi et al (2010)  Anxiety, depression and health-related quality of life in those injured by landmines, Ilam, Islamic Republic of Iran | Civilians | 2007 | 1980-1988 | 137 | 38.1 | 0.066 | BAI | Iran |
| Karam et al (2014)  Outcome of depression and anxiety after war: a prospective epidemiologic study of children and adolescents | Civilians | 1997 | 1975-1990 | 143 | 11.5 | 0.500 | DICA-R | Lebanon |
| Abbo et al (2013)  Prevalence, comorbidity and predictors of anxiety disorders in children and adolescents in rural north-eastern Uganda | Civilians |  | 1986-2006 | 1587 |  | 0.537 | MINI | Uganda |
| Roberts et al (2019) Mental health care utilisation among internally displaced persons in Ukraine: results from a nation-wide survey | Civilians | 2016 | 2014 | 2203 |  | 0.681 | GAD-7 | Ukraine |

Abbreviations: State Trait Anxiety Inventory – STAI; Hopkins Symptom Checklist-25 – HSC-25; Beck Anxiety Inventory – BAI; Diagnostic and Statistical Manual of Mental Disorders III-Revised – DSM-III-R; Diagnostic and Statistical Manual of Mental Disorders IV-Revised – DSM-IV-R; Hospital Anxiety and Depression Scale – HADS; International Classification of Diseases-10 – ICD-10; Mini International Neuropsychiatric Interview – MINI; Hamilton Scale for Anxiety – HAS; General Anxiety Disorder-7 – GAD-7; Depression and Anxiety Subscales of the Symptoms Checklist-90 – DASSC-90;

Summary of PTSD references

| Full reference | Study population | Survey year | Years of war/conflict | Sample size | Mean age | Proportion of females | Tools used | Country |
| --- | --- | --- | --- | --- | --- | --- | --- | --- |
| Donoho et al (2018)  Depression among military spouses: Demographic, military, and service member psychological health risk factors | Civilians | 2011 | Multiple | 9038 |  | 0.87 | PCL-C | United States |
| Ikin et al (2010)  Comorbidity of PTSD and depression in Korean War veterans: prevalence, predictors, and impairment | Military |  | 1950-1953 | 5352 | 75.0 |  | PCL-C | Australia |
| Ikin et al (2007)  Anxiety, post-traumatic stress disorder and depression in Korean War veterans 50 years after the war | Military | 2005 | 1950-1953 | 6122 | 75 |  | PCL-C | Australia |
| Long et al (1996)  Prevalence of posttraumatic stress disorder, depression and anxiety in a community sample of New Zealand Vietnam War veterans | Military |  | 1955-1975 | 756 | 50.0 |  | Mississippi Scale | New Zealand |
| Khateri et al (2017)  Mental health status following severe sulfur mustard exposure: a long-term study of Iranian war survivors | Civilians |  | 1980-1988 | 350 | 45.2 |  | DSM-IV-R | Iran |
| Hashemian et al (2006)  Anxiety, depression, and posttraumatic stress in Iranian survivors of chemical warfare | Civilians | 2004 | 1980-1988 | 153 | 45 |  | PTSD Scale | Iran |
| Marshall et al (2005)  Mental Health of Cambodian Refugees 2 Decades After Resettlement in the United States | Civilians | 2005 | 1955-1975, 1978-1989 | 490 | 52 | 0.61 | HTQ | United States |
| Husain et al (2011)  Prevalence of war-related mental health conditions and association with displacement status in postwar Jaffna District, Sri Lanka | Civilians | 2009 | 1995 | 1409 | 40.2 | 0.675 | HTQ | Sri Lanka |
| Acarturk et al (2021)  Prevalence and predictors of common mental disorders among Syrian refugees in Istanbul, Turkey: a cross-sectional study | Civilians | 2018 | 2011-present | 1678 |  | 0.516 | PCL-C | Syria |
| Elbedour et al (2007)  Post-traumatic stress disorder, depression, and anxiety among Gaza Strip adolescents in the wake of the second Uprising (Intifada) | Civilians |  | 2000-2005 | 229 | 17.1 | 0.472 | PTSD-I | Gaza Strip |
| Somasundaram et al (1994)  War trauma in a civilian population | Civilians | 1991 | 1995 | 98 |  | 0.357 | DSM-III-R | Sri Lanka |
| Servan-Schreiber et al (1998)  Prevalence of posttraumatic stress disorder and major depressive disorder in Tibetan refugee children | Civilians | 1997 | 1951 | 61 |  | 0.508 | DSM-IV-R | Tibet |
| Thomas et al (2010)  Prevalence of mental health problems and functional impairment among active component and National Guard soldiers 3 and 12 months following combat in Iraq | Military | 2004 | Multiple | 5609 |  | 0.039 | PCL-C | United States |
| Kroll et al (2011)  Psychoses, PTSD, and depression in Somali refugees in Minnesota | Civilians | 2009 | 1991-present | 600 |  | 0.535 | DSM-IV-R | United States |
| Steel et al (2002)  Long-term effect of psychological trauma on the mental health of Vietnamese refugees resettled in Australia: a population-based study | Civilians |  | 1955-1975 | 1161 | 41.0 | 0.590 | ICD-10 | Australia |
| Hasanović et al (2008)  Post traumatic stress disorder, depression and anxiety among family medicine residents after 1992-95 war in Bosnia and Herzegovina | Civilians | 2004 | 1992-1995 | 78 |  | 0.846 | HTQ | Bosnia and Herzegovina |
| Summerfield et al (1991)  Low intensity' war and mental trauma in Nicaragua: a study in a rural community | Civilians | 1989 | 1978-1979 | 43 |  | 0.512 | GHQ | Nicaragua |
| Engdahl et al (1991)  Comorbidity of psychiatric disorders and personality profiles of American World War II prisoners of war | Military |  | 1939-1945 | 62 |  |  | DSM-III-R | United States |
| Hovens et al (1994)  Posttraumatic stress disorder in male and female Dutch Resistance veterans of World War II in relation to trait anxiety and depression | Military | 1986 | 1939-1945 | 824 |  | 0.175 | DSM-III-R | Netherlands |
| Toomey et al (2018)  Mental health of US Gulf War veterans 10 years after the war | Military | 1991 | 1990-1991 | 1061 | 38.9 | 0.22 | PCL-C | United States |
| Hauff et al (1993)  Vietnamese boat refugees: the influence of war and flight traumatization on mental health on arrival in the country of resettlement. A community cohort study of Vietnamese refugees in Norway | Civilians | 1982 | 1955-1975 | 145 | 26.0 | 0.214 | DSM-III-R | Norway |
| Thabet et al (2002)  Emotional problems in Palestinian children living in a war zone: a cross-sectional study |  |  | 1948-present | 91 |  |  |  | Palestine |
| Scholte et al (2004)  Mental health symptoms following war and repression in eastern Afghanistan | Civilians | 2003 | 1979-1989 | 1011 |  | 0.55 | HTQ | Afghanistan |
| Başoglu et al (2005)  Psychiatric and cognitive effects of war in former Yugoslavia: association of lack of redress for trauma and posttraumatic stress reactions | Civilians | 2000 | 1991-2001 | 1358 | 39 | 0.41 | DSM-IV-R | Yugoslavia |
| Cardozo et al (2005)  Report from the CDC: mental health of women in postwar Afghanistan | Civilians | 2002 | 1979-1989 | 695 |  | 0.609 | HTQ | Afghanistan |
| Lapierre et al (2007)  Posttraumatic stress and depression symptoms in soldiers returning from combat operations in Iraq and Afghanistan | Military | 2005 | 1979-1989 | 4089 | 26.4 | 0.06 | Structured Interview for PTSD | United States |
| Al-Jawadi et al (2007) Prevalence of childhood and early adolescence mental disorders among children attending primary health care centers in Mosul, Iraq: a cross-sectional study | Civilians | 2007 | 2003-2011 | 3079 |  | 0.45 | DSM-III-R | Mosul |
| Roberts et al (2009)  Post-conflict mental health needs: a cross-sectional survey of trauma, depression and associated factors in Juba, Southern Sudan | Civilians | 2007 | 1983-2005 | 1242 | 33 | 0.507 | HTQ | Sudan |
| Kashdan et al (2009)  Post-traumatic stress disorder, social anxiety disorder, and depression in survivors of the Kosovo War: experiential avoidance as a contributor to distress and quality of life | Civilians | 2006 | 1998-1999 | 164 |  |  | LSC-R | Kosovo |
| Neria et al (2010)  A longitudinal study of posttraumatic stress disorder, depression, and generalized anxiety disorder in Israeli civilians exposed to war trauma | Civilians | 2009 | 2008-2009 | 134 | 24 | 0.84 | PTSD Inventory | Israel |
| Ginzburg et al (2010)  Comorbidity of posttraumatic stress disorder, anxiety and depression: a 20-year longitudinal study of war veterans | Military | 1983 | Multiple | 664 |  |  | PTSD Inventory | Israel |
| Gelkopf et al (2013)  A longitudinal study of changes in psychological responses to continuous terrorism | Civilians | 2002 | Multiple | 153 | 38.2 | 0.542 | DSM-IV-R | Israel |
| Bleich et al (2006) Mental health and resiliency following 44 months of terrorism: a survey of an Israeli national representative sample | Civilians | 2004 | Multiple | 501 |  |  | DSM-IV-R |  |
| Vazan et al (2013) Substance use and other mental health disorders among veterans returning to the inner city: prevalence, correlates, and rates of unmet treatment need | Military | 2011 | Multiple | 269 |  | 0.122 | PTSD Checklist | United States |
| Vaughan et al (2014)  Prevalence of mental health problems among Iraq and Afghanistan veterans who have and have not received VA services | Military | 2010 | Multiple | 913 |  | 0.11 | PTSD Checklist | United States |
| Dursa et al (2019)  Health Status of Female and Male Gulf War and Gulf Era Veterans: A Population-Based Study | Military | 2012 | 1990-1991 | 8104 |  | 0.194 | PTSD Checklist | United States |
| Roberts et al (2019) Mental health care utilisation among internally displaced persons in Ukraine: results from a nation-wide survey | Civilians | 2016 | 2014 | 2203 |  | 0.681 | PCL-C | Ukraine |
| Jayuphan et al (2020)  Mental health problems from direct vs indirect exposure to violent events among children born and growing up in a conflict zone of southern Thailand | Civilians | 2015 | 2004-2021 | 941 | 12.3 | 0.525 | CR-IES | Thailand |
| Kakaje et al (2021) Mental disorder and PTSD in Syria during wartime: a nationwide crisis | Civilians | 2019 | 2011-present | 1951 |  | 0.788 | SPTSS | Syria |
| Farhood et al (2012)  Prevalence and predictors for post-traumatic stress disorder, depression and general health in a population from six villages in South Lebanon | Civilians | 2009 | 1985-2000 | 632 |  |  | HTQ | Lebanon |
| Fox et al (2000)  The Sierra Leonean refugee experience: traumatic events and psychiatric sequelae | Civilians |  | 1991-2002 | 55 | 31.3 | 0.491 | HTQ | West Africa |
| Ayazi et al (2014) Association between exposure to traumatic events and anxiety disorders in a post-conflict setting: a cross-sectional community study in South Sudan | Civilians |  | 1983-2005 | 1200 |  | 0.436 | HTQ | South Sudan |
| Thapa et al (2005) Psychological distress among displaced persons during an armed conflict in Nepal | Civilians | 2003 | 1996-2006 | 290 | 40.9 | 0.390 | PCL-C | Nepal |
| Yaacoub et al A (2020) Posttraumatic stress disorders and depression among male inpatient adults involved in the Lebanese war | Civilians | 2016 | 2016 | 31 | 42.6 | 0 | MINI | Lebanon |
| Yaacoub et al B (2020) Posttraumatic stress disorders and depression among male inpatient adults involved in the Lebanese war | Military | 2016 | 2016 | 31 | 42.6 | 0 | MINI | Lebanon |

Abbreviations: Post-traumatic Stress Disorder – PTSD; PTSD Checklist-Civilian Version – PCL-C; Diagnostic and Statistical Manual of Mental Disorders IV-Revised – DSM-IV-R ; Harvard Trauma Questionnaire – HTQ; PTSD Interview – PTSD-I; Diagnostic and Statistical Manual of Mental Disorders III-Revised – DSM-III-R; General Health Questionnaire – GHQ; Life Stressor Checklist-Revised – LSC-R; Children’s Revised Impact of Events Scale – CR-IES; Screen for Post-Traumatic Stress Symptoms (SPTSS); Mini International Neuropsychiatric Interview – MINI
